# Investigation of Strength Properties for Concrete Containing Fine-Rubber Particles Using UPV

**DOI:** 10.3390/ma15103452

**Published:** 2022-05-11

**Authors:** Yeol Choi, Il-Hyun Kim, Hyeon-Jin Lim, Chang-Geun Cho

**Affiliations:** 1 School of Architecture, Kyungpook National University, Daegu 41566, Korea; choiyeol@knu.ac.kr (Y.C.); kih328@knu.ac.kr (I.-H.K.); 2 Department of Architectural Engineering, Chosun University, Gwangju 61452, Korea; jericho0220@naver.com

**Keywords:** fine-rubber particle, rubber-concrete, UPV, non-destructive test, toughness, strength property

## Abstract

Since the early 1990s, many studies were conducted to utilize waste tires as a replacement for natural coarse and fine aggregates in concrete, known as rubberized concrete or rubber-concrete. In this paper, an experimental study was performed on the strength properties of concrete containing fine-rubber particles as a replacement of fine aggregate, using destructive and non-destructive tests. Ultrasonic pulse velocity (UPV) tests were used to evaluate the strength property of rubber-concrete as a non-destructive test. Compressive and splitting tensile strengths were determined for four different volume contents of fine-rubber particles and exponential equations were proposed for the relationship between compressive, splitting tensile strength and the UPV of rubber-concrete, respectively. With the limited conditions in this paper, it found that UPV tests could also be used to estimate the compressive and tensile strengths of rubber-concrete, that are used in other types of concrete.

## 1. Introduction

Over the years, the treatment of waste tires has been one of the huge environmental concerns in many countries. In the research field of concrete, research has been conducted regarding the waste tire as a replacement for natural aggregates in concrete, known as rubberized concrete or rubber-concrete. A number of studies show that the mechanical properties of rubber-concrete, such as compressive and tensile strengths, can be used for certain specified engineering applications [1,2,3]. To evaluate the mechanical properties of rubber-concrete, normally destructive tests were used. In addition to destructive tests, non-destructive tests can also be used to investigate mechanical properties, setting, hardening and the durability of rubber-concrete. As a non-destructive test, the ultrasonic pulse velocity (UPV) test was introduced in the mid 1940′s for concrete testing. Since then, UPV testing has been widely used to evaluate mechanical properties, defects and the deterioration of concrete. [4,5]. In particular, the relationship between the strength and the UPV value of concrete was investigated by many researchers.

Rao et al. [6] presents some experimental results of UPV testing conducted on roller compacted concrete (RCC), containing ground granulated blast furnace slag (GGBS) as the mineral admixture. Test results showed that, when the compressive strength and UPV values of RCC mixed with GGBS were pooled together, the relationships were exponential and the regression coefficients (R^2^) were different for each replacement level of GGBS. The relationship between the compressive strength and UPV of RCC can be used to estimate the strength of concrete at any age and for all replacement levels of GGBS mixed in RCC.

Demirboga et al. [7] reported the relationship between UPV values and compressive strength of concrete that had high-volume fly ash (FA), blast furnace slag (BFS) and FA+ BFS as the mineral admixtures in replacement for Portland cement (PC), respectively. The relationship between compressive strength and UPV values was determined at the age of 3-, 7-, 28- and 120-days, respectively. Both the compressive strength and UPV values were very low for all the levels of mineral admixtures at an early age, especially for the concrete containing fly ash. However, with the increase in the curing age, both the compressive strength and UPV values in all cases of concrete were increased. The relationship between UPV value and compressive strength was expressed by an exponential equation for FA, BFS and FA + BFS, respectively. However, constants of each exponential equation were different for each mineral admixture and at each level of replacement for Portland cement.

Turgut [8] presented the relationship between concrete strength and UPV values tested from several core specimens of concrete that were taken from different reinforced concrete (RC) structures with different ages and unknown ratios of concrete mixtures. In addition, an interaction function was adopted so as to find the relationship between concrete’s strength and UPV values from the earlier laboratory research on concrete specimens with various mixture ratios. By processing the interaction between these data sets, the best fitting formula for the relationship between concrete strength and UPV values was obtained. Authors introduced a general formula between concrete strength and UPV values without consideration of concrete’s mixture ratios. The presented formula was used to find out concrete strength in existing concrete structures, with or without records of concrete mixing ratios.

Kewalramani et al. [9] reported numerous attempts to use UPV as a measure of compressive strength of concrete, based on conventional computational techniques such as a multiple regression analysis. The authors utilized artificial neural networks (ANN) that could predict the compressive strength based on UPV and the weight of concrete specimens. This study incorporated two different shapes and sizes of concrete specimens for two different concrete mixtures M20 and M30, and a predictive comparison was made with a multiple regression analysis. The prediction using ANN showed a good degree of coherency with experimentally evaluated compressive strength for both shapes and sizes of concrete specimens.

Fawzy et al. [10] presented a review of the properties and applications of rubberized concrete for structural members. The authors found that the increase of rubber content in concrete resulted in a decrease of workability and flowability, and that rubberized concrete showed a lower density than that of ordinary concrete. They also reported that rubberized concrete showed lower compressive and flexural strength in comparison with ordinary concrete.

Gregori et al. [11] predicted the compressive strength of rubberized concrete using support vector machine (SVM) and Gaussian process regression (GPR) models, respectively. The authors used data of compressive strength from previous research results and derived equations of two strength reduction factors (SRF) to predict the compressive strength of rubberized concrete in terms of cement content, fine and coarse aggregate replacement percentages, water/cement ratio, and so on. They concluded that the GPR model was more effective than that of the SVM to estimate the compressive strength of rubberized concrete.

Mohammed et al. [12] investigated the fresh and hardened properties of crumb rubber containing concrete, using UPV and rebound hammer tests as a non-destructive test, respectively. According to the test results, the UPV value slightly decreased as the crumb rubber replacement increased upwards at 3, 7 and 28 days, and the UPV test showed more realistic results for evaluating the rubberized concrete mixtures than the rebound hammer test.

A number of works were carried out to evaluate the relationship between UPV values and strength for various types of concrete. However, there were not many investigations to find the relationship between UPV values and the strength of rubber-concrete at this time. In this study, the mechanical properties of concrete containing fine-rubber particles are investigated, using destructive and non-destructive tests for four different contents of fine-rubber particles varying with temperature. The result of this study intends to estimate the strength properties of rubber-concrete using non-destructive test, and will be provided for use for structural applications of rubber-concrete.

## 2. Experimental Program

In this paper, an experimental program was designed to evaluate strength properties of concrete containing fine-rubber particles as a replacement for fine aggregate. Destructive testing and UPV, as non-destructive testing methods, were conducted for hardened concrete under room and high temperatures at 14, 28, and 56 day curing time [13].

### 2.1. Materials

**Figure 1:**
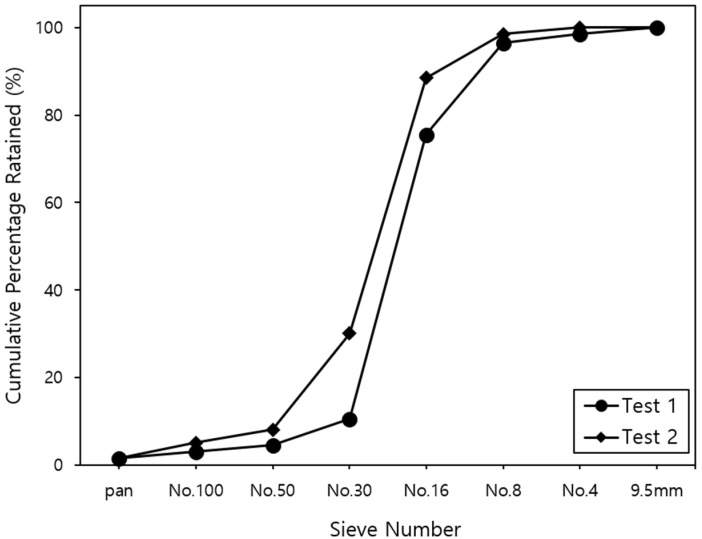
Gradation curves for the used fine-rubber particles.

The cement used in this study was an ordinary Portland cement (type I/II in Korea), which met the Korean standards KSL5201 with a specific gravity of 3.15. The used coarse aggregate has a 19 mm maximum size of crushed stone with a specific gravity of 2.64. The fine aggregate has a specific gravity of 2.56 and a fineness modulus of 2.42 of natural sand. The used waste tires’ rubber-particles, as a replacement for fine aggregate in this study, were manufactured from automobile tires by mechanical shredding. The used rubber particle has an average of 1.0 mm in size, and has a specific gravity of 0.83. The gradation curve of fine-rubber particles in this study was determined based on the KS F 2502 method, and the gradation curves of fine-rubber particles are shown in Figure 1.

### 2.2. Mix Proportion and Specimen

The plain and rubber-concrete specimens were made by a rotary mixer with the following stages: coarse and fine aggregates were mixed for approximately 1 min, and then cement and fine-rubber particles were added in the mixer and mixed for an additional 2~3 min. Mixing continued and then about 50% of the total water was added. Finally, the remaining water was added, and the mixing continued for an additional 4~5 min. In this study, 0%, 10%, 20% and 30% of fine-rubber particle contents were used as a replacement for fine aggregate in concrete, respectively. The details of the mix numbers and mix proportions for the specimens are shown in Table 1. The mix numbers given in Table 1 are represented by the content of the used fine-rubber particles. For example, RPC 10 means that a concrete specimen has 10% of fine-rubber particle content as a replacement for fine aggregate.

Concrete specimens were made using a plastic mold of 100 mm in diameter and 200 mm in height. The casting of specimens both in plain concrete and rubber-concrete were made in a laboratory at room temperature. The used specimens were demolded approximately 24 h after their casting, and then cured in a water tank until the time of testing with an average temperature of 20 ± 1.5 °C. A total of 108 specimens of plain concrete and rubber-concrete were made for UPV test, as well as compressive and splitting tensile tests.

### 2.3. Test Methods

**Figure 2:**
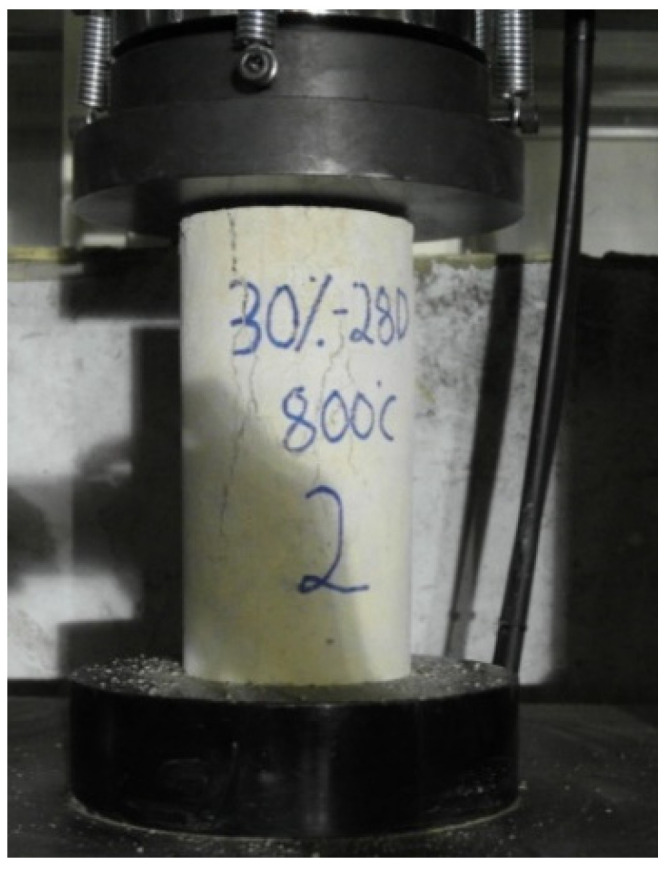
Compression test.

**Figure 3:**
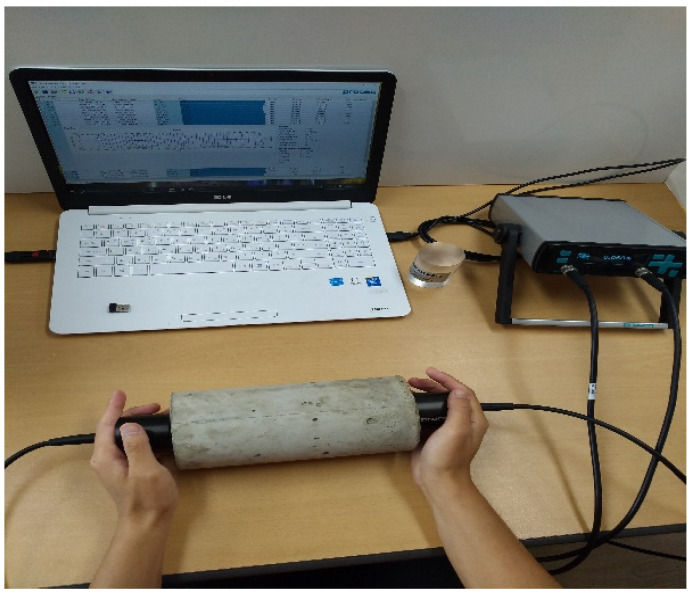
Direct measurement of UPV test.

To evaluate the strength properties of concrete containing fine-rubber particles, compression, splitting tension and ultrasonic pulse velocity tests were carried out for hardened concrete specimens at 14, 28 and 56 days. A total of 54 concrete specimens of plain concrete and rubber-concrete were used for the compression test, in accordance with the Korean standards KSF 2405 (2009). The loading control in a universal testing machine (UTM) was applied at a rate of 0.02 mm/sec with a preload of about 200 N. The peak load and the load–axial displacement in the UTM were recorded during the test using an acquisition system. The compressive strength of each specimen was evaluated at 14, 28 and 56 days, as shown in Figure 2. In general, the splitting tensile test is a relatively simple method to evaluate the tensile strength of concrete, and seems to provide reliable tensile strength of concrete compared to the direct tension test that does not give suitable outcomes on the post-crack behavior of concrete. Therefore, a splitting tensile test was selected for determining the tensile strength of rubber-concrete in this study. The splitting tensile test was carried out according to the Korean standards KSF 2423 (2016). The load in UTM was applied at a rate of 0.01 mm/sec with a preload of about 50 N. As a non-destructive test, a UPV test was used for the concrete specimens to measure the velocity of wave transmission. In general, the UPV test is performed by direct measurement (opposite faces) or indirect measurement (along the surfaces) [14]. In this study, direct measurements were carried out using a concrete specimen with the dimension of 100 mm in diameter and 200 mm in height. A digital indicator tester (PUNDIT) was used as it consisted of two transducers, i.e., one receiver head of 54 kHz ± 5 kHz with bandwidth < 10 kHz and one transmitter. An average of three direct measurements was made in the longitudinal direction of concrete specimens, as shown in Figure 3.

## 3. Results and Discussion

### 3.1. Compressive Test

**Figure 4:**
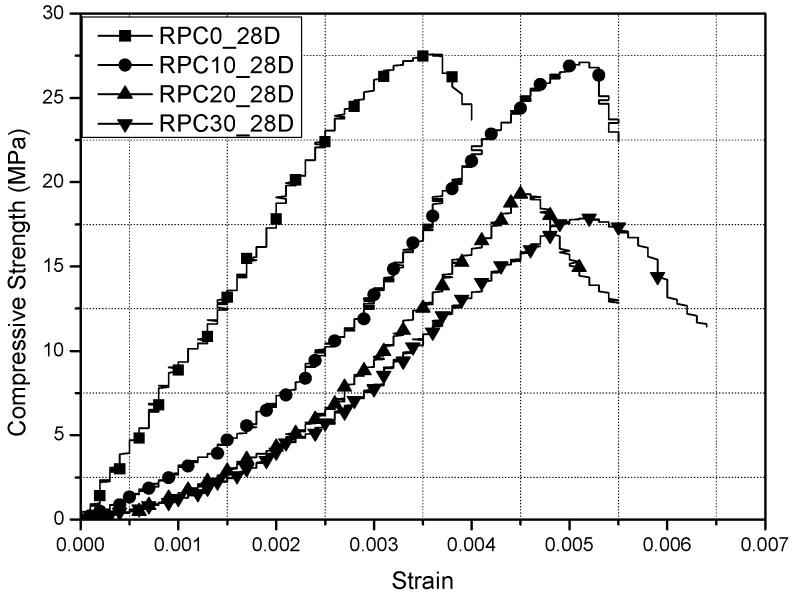
Typical stress–strain curves of rubber-concrete.

The test result is based on the average of three cylindrical specimens tested in each mix. Compressive strengths of the cylindrical specimens were evaluated for ages of 14, 28 and 56 days under room and high temperatures. For each specimen, a stress–strain curve was measured, and a typical stress–strain curve of concrete with different replacement for fine-rubber particles for 28 days is shown in Figure 4. Specimens of rubber-concrete showed a somewhat longer deformation at the peak load under compression compared to plain concrete. From Figure 4, it is found that the higher content of rubber particles (20 and 30% replacement) showed more load carrying beyond the peak load compared to the plain concrete. This result actually came from the bridge role of cracks by the imbedded rubber particles. Further investigation is needed to verify the influence of rubber particles compared to plain concrete.

In general, toughness of the material can be calculated by measuring the area under the typical stress–strain curve, up to 80% of the ultimate stress in the post-peak region [14]. In other words, the toughness index is determined as a ratio between the area under the stress–strain curve up to 80% of the peak stress in the post-peak (*T*_80%_) and the area under the stress–strain curve up to the peak stress (*T*_100%_). Thus, the toughness index, *T_i_*, is expressed as follows:

(1) $$T_{i}=\frac{T_{80\%}}{T_{100\%}}$$

The toughness index of rubber-concrete obtained in this study is presented in Table 2. The toughness indices of rubber-concrete showed slightly higher values than those of the plain concrete. The toughness index of rubber-concrete showed gradual increment as the fine-rubber content increased up to 30% of replacement. The toughness index found here may be compared with the values obtained by Khaloo [14]. Khaloo [14] determined toughness indices from rubber particle replacement of 0 to 50% as coarse and fine aggregates. He suggested that the highest toughness index was found for the concrete containing 25% of the total aggregate volume. The maximum toughness index from this investigation was exhibited at 30% replacement for fine aggregate.

The average compressive strengths of rubber-concrete for curing time and rubber particle content are presented with standard deviations in brackets as shown in Table 3. It is observed that the use of rubber particles in concrete decreased compressive strength at the age of 14, 28, and 56 days. As expected, the mix with higher rubber particles showed the higher reduction in compressive strength. The rubber-concrete with 10, 20 and 30% replacement for fine aggregate showed a decrease in compressive strength of about 12, 32 and 38% compared to plain concrete (RPC 0) for 28 days, respectively. Rubber-concrete also showed a gradual increase of compressive strength with increasing of curing time, as presented in plain concrete. The highest increasing rate of compressive strength from 14 days to 28 days was found at RPC 10. The highest increasing rate of compressive strength from 28 days to 56 days was found at RPC 30. In this study, it may be concluded that the rubber particles up to 10% can be reasonably used for structural applications, due to having sufficient compressive strength at 28 days. Aiello [2] reported that the replacement for coarse aggregate up to 20% could provide acceptable compressive strength for structural applications. This study, however, clearly suggests the need for further research on the compressive strength of rubber-concrete so that it can be successfully used as one of the structural materials.

The average compressive strengths of plain and rubber-concrete for high temperatures are presented with standard deviations in brackets, as shown in Table 4. The cylindrical specimens at 28 days were heated at temperatures from 200 to 800 °C with intervals of 200 °C [15]. All specimens were exposed gradually up to a target temperature in the furnace, and exposed to the target temperature for about 1 h. After heating, compressive strength was determined by the average of three cylindrical specimens for each mix. It was observed that the mix with the higher rubber particles showed relatively higher reduction in compressive strength for each high temperature. The rubber-concrete with 10, 20, and 30% replacement for fine aggregate showed a decrease in compressive strength of approximately 22.19, 16.75, and 25.48% at 200 °C, respectively. Compressive strength of all rubber-concretes after being exposed to 800 °C decreased dramatically by as much as 83%, compared to that at room temperature.

### 3.2. Splitting Tensile Strength (STS)

The average splitting tensile strength for curing time and rubber particle content is shown in Table 5. The splitting tensile strength of rubber-concrete increased when the curing time was increased. It is observed that the use of rubber particles decreased the splitting tensile strength at 14, 28 and 56 days, compared to plain concrete. As expected, the mix with higher rubber particles showed a higher reduction in splitting tensile strength. The rubber-concrete with 10, 20 and 30% replacement for fine aggregate showed a decrease in splitting tensile strength of about 9, 30 and 37% compared to the plain concrete (RPC 0) at 28 days, respectively. The decreasing rate of splitting tensile strength shows a similar rate to that of compressive strength.

The average splitting tensile strength of rubber-concrete for high temperatures is also shown in Table 6. The specimens at 28 days were heated at temperatures from 0 to 800 °C with intervals of 200 °C. It was observed that the mix with higher rubber particles showed the higher reduction in compressive strength under a constant high temperature. The rubber-concrete showed a rapid reduction of splitting tensile strength after being exposed to 800 °C in this study. It was also observed that the mix with higher rubber particles exhibited the lower residual splitting tensile strength. The residual splitting tensile strength for 0, 10, 20 and 30% replacement for fine aggregate is shown as 16.75, 14.12, 13.80, and 13.44%, respectively.

In order to find the effect of rubber particles on the strength of rubber-concrete, the relationship between compressive and splitting tensile strength was investigated. The ratio of splitting tensile strength to compressive strength for rubber concrete showed a similar value to that of plain concrete. At the age of 28 days, the ratio was 16.14, 16.20, and 15.96% for 10, 20, and 30% of rubber particle replacement for fine aggregate, respectively. The ratio was 16.57, 15.80, and 16.32% at 56 days, respectively. The relationship between compressive and splitting tensile strength after being exposed to high temperature was also investigated. There was no significant difference compared to plain concrete for room and high temperatures. The ratio at 200 °C was 16.15, 16.44, and 15.68% for 10, 20, and 30% of rubber particle replacement for fine aggregate, respectively. The ratio was slightly decreased for 400 °C and 800 °C.

### 3.3. Ultrasonic Pulse Velocity (UPV)

The UPV test was carried out to measure the velocity of wave transmission for specimens of plain concrete and rubber-concrete at 14, 28 and 56 days. The UPV test was measured at a very small deformation of specimens, while strength was measured at the failure state of specimens.

**Figure 5:**
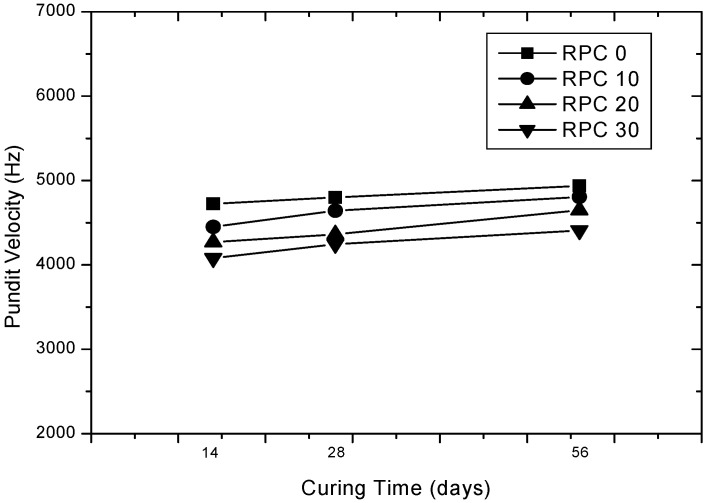
UPV for curing time.

**Figure 6:**
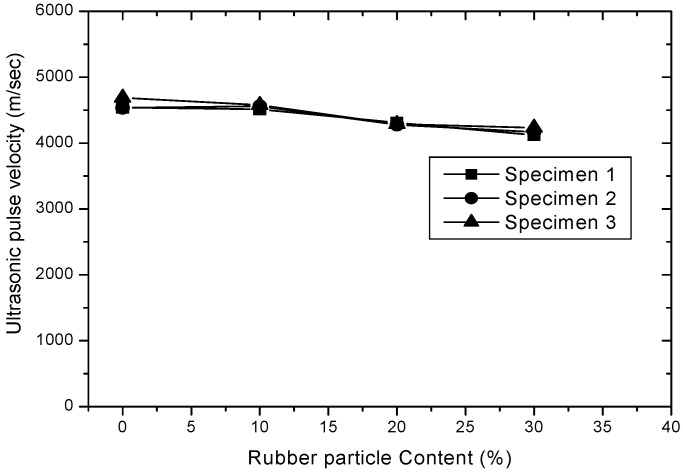
UPV for rubber particle content.

The average UPV values of rubber-concrete for curing time and contents of rubber particles are shown in Figure 5 and Figure 6, respectively. Test results show that the UPV values of both plain concrete and rubber-concrete are increased almost linearly as the curing time increased. The observed UPV values of 0%, 10%, 20% and 30% of rubber particle contained concrete ranged from 4724 m/s to 4936 m/s, 4450 m/s to 4805 m/s, 4270 m/s to 4650 m/s, 4079 m/s to 4408 m/s, respectively. The IS 13311 (part 1; 1992) suggested that a UPV of greater than 4500 m/s is considered to be excellent quality, 3500~4500 m/s is considered to be good quality, 3000~3500 m/s is considered medium quality and less than 3000 m/s is considered to be doubtful quality concrete [6]. Based on the IS 13,311 (part 1), it can be concluded that all of the rubber-concretes at 28 days are shown as an excellent quality. In addition, the average increment of UPV value at 28 and 56 days compared to 14 days are 1.58~4.32% and 4.48~8.89%, respectively. The UPV values of both plain and rubber-concrete are almost linearly decreased when rubber particle contents are increased from 0 to 30%. Compared to plain concrete, the average reduction of UPV value ranged from 5.80 to 13.65% at 14 days, 3.25 to 11.52% at 28 days and 2.65 to 10.69% at 56 days, respectively.

The average UPV measurements of plain and rubber-concrete exposed to 0, 200, 400 and 800 °C are shown in Table 7. As expected, the UPV of both plain concrete and rubber-concrete steadily decreased as the exposure temperature increased. Reductions were ranged approximately 7.18–12.96% at 200 °C, 21.72–40.15% at 400 °C, and 73.72–85.72% at 800 °C. In particular, the UPV values of both concretes were dramatically decreased at 800 °C, and were 73.72, 84.26, 85.73 and 85.44% for RPC 0, 10, 20 and 30%, respectively. This result showed a very similar reduction of compressive and splitting tensile strength of plain concrete and rubber-concrete after exposure to high temperatures, as shown earlier. Therefore, it can be concluded that UPV measurements can be used to estimate the strength properties of concrete exposed to high temperatures. In addition, it was found that decreasing the rate of UPV at 400 °C was 21.73, 27.64, 26.57 and 40.15% for RPC 0, 10, 20 and 30%, respectively. From the test results, UPV of concrete with higher rubber content showed a greater decreasing rate.

## 4. Relationship between Strength and UPV

**Figure 7:**
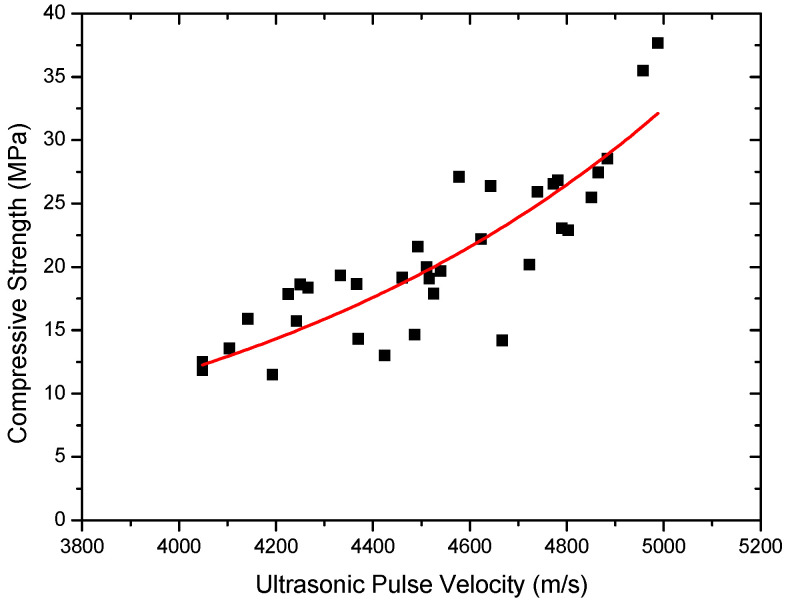
Relationship between UPV and compressive strength.

Until now, a number of researchers have developed theoretical models for the estimation of relationship between the UPV measurement and the destructive test of compressive strength. It is believed that UPV measurement can also be correlated to compressive and tensile strengths. However, this relationship can be affected by many factors, such as types of aggregates, W/C ratios, curing time, specimen sizes and the testing methods used. Therefore, the UPV value can also be used to estimate compressive and tensile strengths of rubber-concrete [16,17,18]. In this section, the relationship between the UPV measurements and the strength properties of rubber-concrete will be presented. Many researchers used an exponential relationship between the compressive strength and the UPV measurement for normal concrete [19]. The general form of the exponential relationship was given as:

(2) $$F_{c}=Ae^{\left(B\right)V_{p}}$$

where, *F_C_* is compressive strength, *A* and B are empirical parameters determined by the regression analysis and *V_p_* is the ultrasonic pulse velocity. A correlation between compressive strength and a UPV measurement is shown in Figure 7, with the data obtained from this study. In order to find a compressive strength–UPV measurement relationship, a reliable curve fitting method is applied. The best curve-fitting equation of compressive strength and UPV value of rubber-concrete can be adopted as:

(3) $$F_{c}=0.194e^{\left(0.00102\right)V_{p}}$$

The best curve-fitting equation is plotted in Figure 7 with the obtained data. The R^2^ value was found to be 0.74.

**Figure 8:**
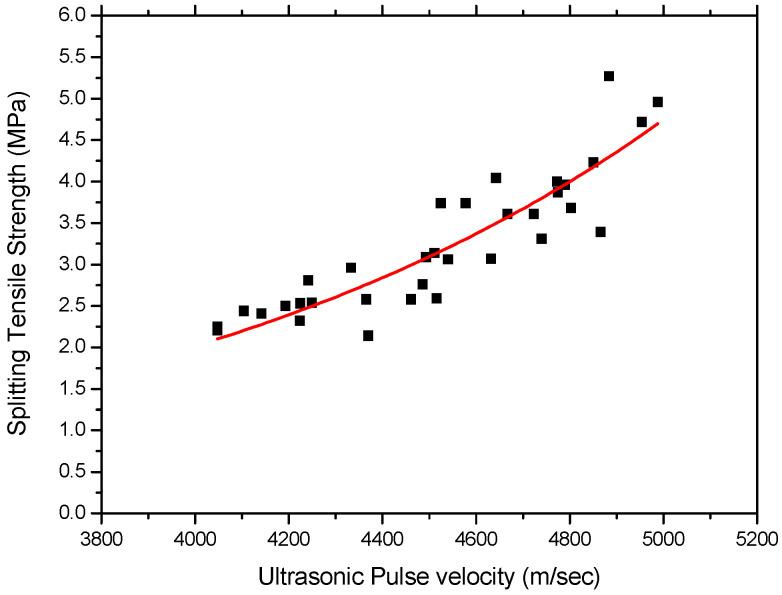
Relationship between UPV and Splitting tensile strength.

A correlation of splitting tensile strength and UPV measurement is shown in Figure 8 with the data obtained from this study. The best curve-fitting equation of splitting tensile strength and UPV value of rubber-concrete can be expressed as:

(4) $$F_{s}=0.065e^{\left(0.00185\right)V_{p}}$$

where, *F_S_* is the splitting tensile strength. The best curve-fitting equation is plotted in Figure 8 with the obtained data, and R^2^ value is found to be 0.79.

It was found that the coefficients of determination (R^2^) obtained in this study are relatively low compared to other different concretes, as shown in Table 8. Most statisticians consider a R^2^ of 0.7 or higher to be reasonable analysis [20]. Therefore, the obtained exponential equation in this study may successfully be used to estimate the relationship between strength properties and UPV measurement on the rubber-concrete.

Table 8 shows some exponential relationships between compressive strength and the ultrasonic pulse velocity waves, with the coefficients of determination R^2^ and specific conditions of concrete. From Table 8, it can be found that each exponential equation obtained in this study shows reasonable results for the estimation of the relationship between UPV and strength for rubber-concrete.

## 5. Conclusions

Based on the experimental and analytical results of concrete containing fine-rubber particles in this study, the following conclusions were made:

1. It is observed that the adding of fine-rubber particles as a replacement for fine aggregates in concrete decreased the compressive and splitting tensile strength at 14, 28, and 56 days. As expected, the mix with higher fine-rubber contents showed a higher reduction both in compressive and splitting tensile strengths;
2. It can be considered that rubber particles as a replacement for fine aggregates in concrete exceeding 20% are not recommended for structural applications, due to the lower compressive strength obtained from destructive tests;
3. However, ultrasonic pulse velocity of concrete containing fine-rubber particles up to 30% replacements of fine aggregates showed excellent qualities based on the IS 13311 (part 1) at 28 days. To verify this result, further investigation is needed under various factors such as types and sizes of rubber, water-cement ratios, curing time, replacement ratios of rubber particles, and so on;
4. Rubber-concrete showed a large reduction in compressive and splitting tensile strengths after being exposed to 800 °C compared to that at room temperature. Further detailed investigations are also needed between temperatures of 400 and 800 °C to verify this result;
5. An exponential relationship between the UPV measurement and the strength of rubber-concrete was proposed. It is concluded that UPV measurements can also estimate the strength of rubber-concrete. However, further investigation is needed under various factors considered in concrete.

## Figures and Tables

**Table 1:** Used mix proportions.

Mix No.	Cement (kg/m^3^)	Water (kg/m^3^)	Coarse Agg. (kg/m^3^)	Fine Agg. (kg/m^3^)	Plasticizer (kg/m^3^)	Amount of Rubber Particle (kg/m^3^)
RPC 0	350	210	1076	697	1.05	0
RPC 10	350	210	1076	627	1.05	23
RPC 20	350	210	1076	558	1.05	46
RPC 30	350	210	1076	488	1.05	69

**Table 2:** Toughness index for rubber-concrete.

Mix No.	*T*_80%_	*T*_100%_	Toughness Index
RPC 0	0.0517	0.0490	1.205
RPC 10	0.0667	0.0548	1.217
RPC 20	0.0400	0.0298	1.344
RPC 30	0.0496	0.0367	1.352

**Table 3:** Compressive strength of rubber-concrete.

Curing Time (Day)	Content	Mix NO. (MPa)			
		RPC 0	RPC 10	RPC 20	RPC 30
14 days	Compressive strength	23.52(2.56)	18.13(3.32)	16.25(1.37)	13.42(3.21)
28 days	Compressive strength	27.00(3.14)	23.79(1.58)	18.33(2.28)	16.60(2.56)
56 days	Compressive strength	33.54(1.87)	24.26(1.28)	21.39(2.45)	17.28(3.05)

**Table 4:** Compressive strength of rubber-concrete for high temperatures.

Temp. (°C)	Content	Mix NO. (MPa)			
		RPC 0	RPC 10	RPC 20	RPC 30
0	Compressive strength	27.00(3.14)	23.79(1.58)	18.33(2.28)	16.60(2.56)
200	Compressive strength	21.76(3.36)	18.51(4.13)	15.26(2.68)	12.37(3.58)
400	Compressive strength	17.51(2.89)	15.20(3.26)	12.61(3.96)	10.40(4.22)
800	Compressive strength	4.67(3.57)	4.03(4.02)	3.08(3.40)	2.68(4.28)

**Table 5:** Splitting tensile strength of rubber-concrete.

Curing Time (Day)	Content	Mix NO. (MPa)			
		RPC 0	RPC 10	RPC 20	RPC 30
14 days	Splitting tensile strength	3.63	2.93	2.54	2.29
28 days	Splitting tensile strength	4.19	3.84	2.97	2.65
56 days	Splitting tensile strength	4.84	4.02	3.38	2.82

**Table 6:** Splitting tensile strength of rubber-concrete for high temperatures.

Temp. (°C)	Content	Mix NO. (MPa)			
		RPC 0	RPC 10	RPC 20	RPC 30
0	Splitting tensile strength	4.19	3.84	2.97	2.65
200	Splitting tensile strength	3.25	2.99	2.51	1.94
400	Splitting tensile strength	2.69	2.30	1.79	1.62
800	Splitting tensile strength	0.59	0.49	0.41	0.34

**Table 7:** UPV value after exposed to high temperatures.

Mix No.	Content	Temperature (°C)			
		0	200	400	800
RPC 0	UPV (m/s)	4630	4202	3550	1232
RPC 10	UPV (m/s)	4492	4187	3306	720
RPC 20	UPV (m/s)	4398	3983	3142	604
RPC 30	UPV (m/s)	4140	3586	2494	624

**Table 8:** Proposed equations with other investigations.

Researcher	Equation	R^2^	Remark
Turgut	$C=1.146e^{\left(0.77\right)V_{p}}$	0.80	Core RC
	$C=0.3161e^{\left(1.03\right)V_{p}}$	0.80	Existing RC
Demirboga	$C=0.0142e^{\left(0.0018\right)V_{p}}$	0.97	FA concrete
	$C=0.0049e^{\left(0.0021\right)V_{p}}$	0.96	BFS concrete
Rao	$C=0.008e^{\left(1.745\right)V_{p}}$	0.91	Plain concrete
	$C=0.003e^{\left(1.934\right)V_{p}}$	0.92	10% GGBS
Present study	$F_{c}=0.194e^{\left(0.00102\right)V_{p}}$	0.74	Rubber-concrete compression
	$F_{s}=0.065e^{\left(0.00185\right)V_{p}}$	0.79	Rubber-concrete splitting tension

## Data Availability

Data sharing is not applicable to this article.
